# Risk factors for ICU mortality in patients with hematological malignancies: a single-center, retrospective cohort study from Turkey

**DOI:** 10.55730/1300-0144.5590

**Published:** 2022-11-01

**Authors:** Gülbin AYGENCEL, Onur GÖKÇE, Merve HAŞİMOĞLU, Nazlıhan BOYACI DÜNDAR, Melda TÜRKOĞLU, Zeynep Arzu YEĞİN, Zübeyde Nur ÖZKURT, Münci YAĞCI

**Affiliations:** 1 Division of Intensive Care Medicine, Department of Internal Medicine, Faculty of Medicine, Gazi University, Ankara, Turkey; 2 Division of Hematology, Department of Internal Medicine, Faculty of Medicine, Gazi University, Ankara, Turkey

**Keywords:** Outcome, prognostic factors, hematological malignancy patients, intensive care unit, acute renal failure, invasive mechanical ventilation, septic shock, SOFA score

## Abstract

**Background/aim:**

Patients with hematological malignancies (HM) often require admission to the intensive care unit (ICU) due to organ failure, disease progression or treatment-related complications, and they generally have a poor prognosis. Therefore, understanding the factors affecting ICU mortality in HM patients is important. In this study, we aimed to identify the risk factors for ICU mortality in our critically ill HM patients.

**Materials and methods:**

We retrospectively reviewed the medical records of HM patients who were hospitalized in our medical ICU between January 1, 2010 and December 31, 2018. We recorded some parameters of these patients and compared these parameters by statistically between survivors and nonsurvivors to determine the risk factors for ICU mortality.

**Results:**

The study included 368 critically ill HM patients who were admitted to our medical ICU during a 9-year period. The median age was 58 (49–67) years and 63.3% of the patients were male. Most of the patients (43.2%) had acute leukemia. Hematopoietic stem cell transplantation (HSCT) was performed in 153 (41.6%) patients. The ICU mortality rate was 51.4%. According to univariable analyses, a lot of parameters (e.g., admission APACHE II and SOFA scores, length of ICU stay, some laboratory parameters at the ICU admission, the reason for ICU admission, comorbidities, type of HM, type of HSCT, infections on ICU admission and during ICU stay, etc.) were significantly different between survivors and nonsurvivors. However, only high SOFA scores at ICU admission (OR:1.281, p = 0.004), presence of septic shock (OR:17.123, p = 0.0001), acute kidney injury (OR:48.284, p = 0.0001), and requirement of invasive mechanical ventilation support during ICU stay (OR:23.118, p = 0.0001) were independent risk factors for ICU mortality.

**Conclusion:**

In our cohort, critically ill HM patients had high ICU mortality. We found four independent predictors for ICU mortality. Yet, there is still a need for further research to better understand poor outcome predictors in critically ill HM patients.

## 1. Introduction

Over the last two decades, therapeutic advances have been significantly beneficial to patients with hematological malignancies (HM). As a result, a growing number of people continue to live with active hematological malignancies. Despite better outcomes, these patients are at risk for life-threatening acute illness due to HM, adverse treatment effects, hematopoietic stem cell transplantation (HSCT) complications [such as acute and chronic graft-versus-host disease (GVHD)], infectious diseases, and decompensation of comorbid conditions. Therefore, the number of critically ill patients requiring intensive care unit (ICU) admission there has been increasing [1]. This patient group still has a high ICU mortality rate, ranging from 25% to 85% [2–4]. However, this rate has recently been declining thanks to broadened ICU admission policies, close collaborations between hematologists and ICU specialists, and improved therapeutic and supportive interventions in ICU practice. Some previous studies have revealed some poor prognostic factors for these patients in the ICU, but some of them are now no longer valid today (such as age, type of HM, presence of neutropenia, etc.) [5–8]. Most of these studies have been from Europe, South and North America. However, very few studies have focused on the prognosis and prognostic factors of these patients admitted to the ICU in Turkey [9–14]. Here, we evaluated prognostic factors associated with ICU outcomes in critically ill HM patients who were admitted to the ICU of a tertiary university hospital in Turkey over a nine-year period.

## 2. Methods

**Figure 1:**
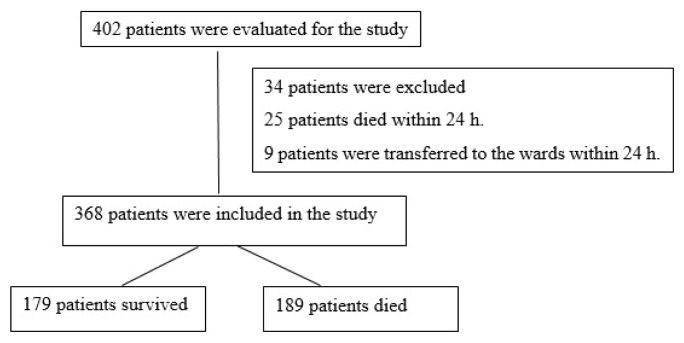
Flow chart of the study.

This retrospective cohort study was carried out in a university hospital in Ankara, Turkey. This hospital is a tertiary referral hospital with 1000 beds, including a general medical intensive care unit (GM-ICU; >30 HM patients admitted per annum) with 9 beds, a hematology intensive care unit (H-ICU; opened in 2014, >80 HM patients admitted per annum) with 4 beds, hematology in-patient clinics with 38 beds, and a bone marrow transplantation (BMT) unit with 8 beds. The BMT unit performs about 70 HSCT procedures per annum. The hematology in-patient clinics care for all types of hematological malignancy patients, and autologous or allogeneic stem cell transplantation is performed in the BMT unit. For this research, we included all HM patients who were nonelectively admitted to the GM-ICU or the H-ICU of this hospital from January 1, 2010 to December 31, 2018. For patients who were admitted to the ICU more than once, we only used their first admission data. Also, we excluded all patients who stayed in the ICU for less than 24 h. Similarly, patients younger than 18 years of age were excluded (Figure 1). We retrospectively reviewed the database to collect patient data. Accordingly, we recorded the following information:

**Factors associated with illness prior to ICU admission:**

○ Demographic data,
○ Comorbidities,
○ Underlying HM diagnosis (acute or chronic leukemia, Hodgkin’s or non-Hodgkin’s lymphoma, multiple myeloma, etc.),
○ HM status (newly diagnosed, refractory/relapse, remission, or terminally ill),
○ Presence and type of HSCT (autologous or allogeneic),
○ Time between first ICU request and ICU admission.

**Factors associated with illness after ICU admission:**

○ Acute Physiology and Chronic Health Evaluation (APACHE) II and Sequential Organ Failure Assessment (SOFA) scores (calculated within 24 h of admission),
○ Reason for ICU admission (e.g., acute respiratory failure, sepsis/septic shock, altered mental status, postoperative care, etc.),
○ Vital signs at ICU admission,
○ Vasopressor and mechanical ventilation support requirements at admission and during ICU stay,
○ Acute kidney injury and renal replacement therapy during ICU stay,
○ Neutropenia at ICU admission,
○ Presence of infection at ICU admission or during ICU stay,
○ Length of ICU stay,
○ Complications during ICU stay (Gastrointestinal-GI bleeding, cardiac complications, etc.),
○ Other treatment related data during ICU stay (usage of blood/blood products, antimicrobials, etc.),
○ ICU mortality.

We also obtained some major laboratory parameters (liver enzymes, creatinine, neutrophil count, hemoglobin, platelets, etc.) within 24 h of ICU admission. The primary outcome was ICU mortality.

This study was performed in line with the principles of the Declaration of Helsinki. Approval was granted by the Ethics Committee of Gazi University (date: 03.08.2021 / no: 2021/734). Since this was a retrospective review of routinely collected data, the ethics committee granted a waiver of informed consent.

### 2.1. Definitions

We used the Third International Consensus Definitions for Sepsis and Septic Shock (Sepsis-3). Accordingly, sepsis was defined as life-threatening organ dysfunction caused by a dysregulated host response to infection. Septic shock was defined as sepsis with persisting hypotension requiring vasopressors to maintain a mean arterial pressure (MAP) of 65 mmHg and having a serum lactate level >2 mmol/L despite adequate volume resuscitation [15]. Acute respiratory failure (ARF) was defined as tachypnea (respiratory rate >30 breaths per minute), clinical signs of respiratory distress (requiring accessory respiratory muscle use or having respiratory muscle exhaustion, etc.), hypoxemia (oxygen saturation <90% or PaO_2_ <60 mmHg on room air), pulmonary infiltrates, and requiring noninvasive or invasive ventilation support [16]. Acute respiratory distress syndrome (ARDS) was defined as acute onset of respiratory failure, bilateral infiltrates on chest radiograph, hypoxemia based on a PaO_2_/FiO_2_ ratio of 300 mmHg, and a lack of evidence of cardiogenic edema [17]. Acute kidney injury (AKI) was defined according to the RIFLE criteria (stages: no risk, risk, injury, failure, loss of function, or end-stage renal disease) [18]. Renal replacement therapy (RRT) was defined continuous venovenous hemofiltration/hemodialysis or intermittent hemodialysis use. Ventilator-associated pneumonia (VAP) was defined as pneumonia developing 48 h or more after mechanical ventilation, characterized by the presence of a new or progressive infiltrate, signs of systemic infection (fever, altered white blood cell count), changes in sputum characteristics, and detection of a causative agent [19]. Neutropenia was defined as having a neutrophil count <1000/mm^3^ [20]. Infection was diagnosed if there was documentation of positive cultures in blood, urine, sputum, endotracheal aspirate, bronchial lavage, wound swabs, or catheter tips, with clinical signs of infection. GI bleeding was suspected when the patients had hematemesis, melena and/or hematochezia, and/or also decrease in hemoglobin level or hemodynamic instability. Diagnosis was confirmed by upper and/or lower endoscopy in patients who could tolerate the procedure.

### 2.2. Statistical analysis

All statistical analyses were performed using the SPSS (Statistical Package for Social Sciences) software, version 22.0 (IBM Corporation, Armonk, NY, USA). We expressed the data as mean (± standard deviation) or median (25–75 percentiles) for continuous variables and as numbers with percentages for categorical variables. We used the chi-squared test and Fisher’s exact test for categorical variables and Student’s t-test or Mann-Whitney U test for continuous variables in univariate analyses. We also performed multivariate analysis with logistic regression analysis to test for associations between certain variables and outcomes. If a variable was associated with the outcome with a p-value <0.05, it was included in the multivariate logistic model. p-value < 0.05 was considered as statistically significant.

## 3. Results

The population for this research consisted of 402 HM patients during the study period. We included only 368 of these patients in our sample (Figure 1). This sample had a median age of 58 (49–67) years, and 63.3% of the patients were male. Two hundred and eighty-one patients were transferred from the hematology wards and 64 from the emergency department. Other patients (23) were transferred from the other intensive care units (8 patients; neurology, neurosurgery or respiratory ICUs) or other in-patient clinics (11 patients; infection disease, medical oncology, general internal medicine or general surgery clinics, etc.) or other hospitals (4 patients). The median length of hospital stay before ICU admission was 5 (0–24) days. The median APACHE II score at admission was 23 (18–27) and the median SOFA score at admission was 8 (6–11). The most frequent HM types were acute leukemia (43.2%) and multiple myeloma (29.3%). 127 patients (34.5%) had newly diagnosed malignancies and 64 (17.4%) were in complete or partial remission. One hundred and fifty-three patients (41.6%) underwent HSCT [autologous in 75 (20.4%), allogeneic in 94 (25.5%), and both in 16 (4.3%)]. The median time from allogeneic HSCT to ICU admission was 210 (48–600) days, and the median time from autologous HSCT to ICU admission was 425 (144.5–1207.5) days. The main reasons for ICU admission were sepsis/septic shock in 277 patients (75.3%) and respiratory failure in 251 (68.2%). The median waiting time for ICU admission was 6 (4–12) hours. The median waiting time was 16 (8–22.5) h before 2014 (before opening separate hematological ICU), whereas it was 5 (3–8) h after 2014. The median length of ICU stay was 6 (4–12) days. Suspected or documented infections at admission were present in 277 patients (75.3%), 215 of which (58.4%) were pulmonary infections. At ICU admission, One hundred and twenty-seven patients (34.5%) had septic shock and received vasopressor agents, 146 (39.7%) were neutropenic, 85 (23.1%) received invasive mechanical ventilation support, 36 (9.8%) had chronic renal failure, and 174 (47.3%) had acute kidney injury (risk, injury, or failure stages according to RIFLE). Table 1 shows the general characteristics of the study patients.

Ninety-seven patients (26.4%) acquired infections during their ICU stay. These included 43 bloodstream/catheter-related bloodstream infections, 66 nosocomial pneumonia/ventilator-associated pneumonia, and 38 urinary tract infections. The most frequent pathogens were the multidrug-resistant *Acinetobacter baumannii* and *Klebsiella pneumoniae*, *Escherichia coli*, and coagulase-negative *Staphylococci*. During ICU stay, 227 patients (61.7%) required invasive mechanical ventilation support, 116 (31.5%) developed AKI, 145 underwent renal replacement therapy (intermittent hemodialysis or continuous hemodiafiltration), and 123 (33.4%) developed septic shock (Table 2).

The overall ICU mortality rate was 51.4% (189 patients). Some of the patients who died (26; 13.8%) were end-stage malignancy patients. However, most of the patients (154; 81.4%) died due to sepsis/septic shock and related multiorgan failure. Cardiac causes (fatal arrhythmias, acute coronary syndrome, etc.) and intracranial causes (intracranial hemorrhage and brain death, etc.) were among the other causes of death (9; 4.8%). According to univariate analyses, APACHE II and SOFA scores at admission, length of ICU stay, waiting time for ICU admission, some laboratory parameters (blood hemoglobin, platelet, leukocyte, urea, sodium, albumin, total bilirubin levels, etc.), the unit before ICU (hematology wards, emergency department, etc.), reason for ICU admission, comorbidities, HM type, HSCT type, invasive mechanical ventilation support requirement at admission and during ICU stay, infection at admission and acquired infections during ICU stay, AKI and renal replacement therapy requirement after ICU admission, septic shock during ICU stay, blood and blood product replacement during ICU stay, and antimicrobial treatments after admission differed significantly between survivors and nonsurvivors. Accordingly, nonsurvivors had higher APACHE II and SOFA scores at admission, waited longer for ICU admission, and stayed longer in ICU. They also had lower hemoglobin, albumin, and platelet levels, and higher blood urea, sodium, alanine aminotransferase, lactate dehydrogenase, and bilirubin levels. Moreover, being transferred from the hematology wards was more common among nonsurvivors. Considering the reasons for ICU admission, respiratory failure and sepsis/septic shock were more common among nonsurvivors. Solid tumors were also more prevalent in nonsurvivors. Regarding HM types, multiple myeloma was more common among survivors. Nonsurvivors received more invasive mechanical ventilation support at admission or during ICU stay, and they had more noninvasive mechanical ventilation failure rates during ICU stay. They had more suspected or detected infections at admission or during ICU stay. They also developed AKI at a higher frequency and underwent more renal replacement therapy during ICU stay. Besides, nonsurvivors had more septic shock and received more blood and blood product replacement during ICU stay. Finally, they received more antimicrobials, particularly antifungal agents, during their ICU stay. Tables 1 and 2 show the results of the univariate analyses and gives a comparison between survivors and nonsurvivors.

**Figure 2:**
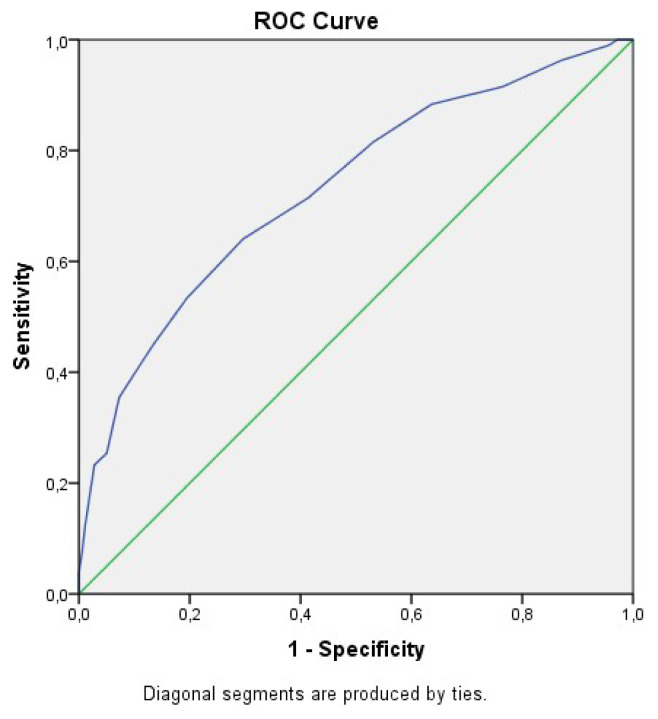
ROC curve for SOFA score at ICU admission (AUC: 0.731, 95% CI 0.680–0.782, p = 0.0001).

We performed multivariate analyses for the aforementioned 18 variables. Accordingly, 4 of these were associated with a marked increase in ICU mortality. These were the SOFA score at admission [OR: 1.281, 95%CI (1.082–1.517), p = 0.004], septic shock during ICU stay [OR: 17.123, 95% CI (4.954–59.183), p = 0.0001], AKI during ICU stay [OR: 48.284, 95% CI (12.232–190.594), p = 0.0001], and invasive mechanical ventilation support requirement during ICU stay [OR:23.118 95% CI (6.577–81.263), p = 0.0001] (Table 3). A SOFA score of 8.5 at admission was a moderate predictor of poor ICU outcome (AUC: 0.731, 95% CI 0.680–0.782, p = 0.0001; sensitivity 64%, specificity 70.4%) (Figure 2).

## 4. Discussion

In this study, we retrospectively evaluated 368 critically ill HM patients for prognostic factors associated with ICU outcome during a 9-year period. The HM patients in our sample who required ICU admission had a high mortality rate (51.4%). We evaluated numerous variables, but only four (high SOFA score at ICU admission, development of septic shock and acute kidney injury during ICU stay, and requirement of invasive mechanical ventilation support during ICU stay) were associated with a poor ICU outcome. This study is one of the few studies presenting ICU outcomes among adult HM patients in Turkey [9–14].

A growing population worldwide, HM patients may require ICU support due to malignancy-related issues and treatment complications. Data shows high ICU and in-hospital mortality rates for this patient group. The mortality rate for HM patients who require ICU admission reportedly ranges from 25% to 85% [2–4]. For our sample, the ICU mortality rate was 51.4%. This rate is similar with most findings in Asian countries and Turkey [8,9–14], although mostly higher than those reported in European and North American countries [21,22]. This difference can stem from various reasons, such as acute disease severity, hematological malignancy type and severity, number of difficult-to-treat cases, variations in ICU admission and discharge criteria, available resources, variations in treatment protocols, and differences in end-of-life decisions.

Here, we observed the changes in our ICU mortality rates during the 9-year study period. Accordingly, the median ICU mortality rate decreased from 78.4% in 2010 to 33.6% in 2018. We can attribute this decrease to an increased experience in care and overall improvements in ICU care and hematological therapies. Also, our hospital established a 4-bed ICU exclusively for these patients in 2014. We showed in a previous study that the presence of a special ICU for these patients reduced ICU mortality [23]. This also allows earlier transfer to the ICU (less waiting time for ICU admission [16 (8–22.5) h before 2014 vs. 5 (3–8) h after 2014, p = 0.0001]), benefitting rescuable patients. Besides, managing more cases of the same type increases clinical experience faster, leading to better outcomes. Similar correlations between case volume and outcomes were described before for specific groups of critically ill patients [24,25].

The literature is replete with studies trying to find out HM patient who would benefit from ICU admission, sometimes with conflicting results [21,22,26]. Several factors such as age, presence of neutropenia, disease type and status have been found controversial in these studies. Some studies have found that patients with active acute myeloid leukemia, advanced age or neutropenia have poor prognosis, whereas we did not find any correlation between these factors and ICU mortality. But recently, a pragmatic approach is suggested to decide. Accordingly, patients who recently began first-line chemotherapy, patients with low-grade HM, and patients with partial remission are always admitted, with a full-code ICU status. In this group, treatment may be discontinued after 3 to 5 days if there is deterioration or no improvement [27]. Patients who are already chronically debilitated in the ward or for whom there is no further life-prolonging causal treatment are not admitted [26,28]. Most research in this regard is from developed countries, where decision-making is easier for these patients. This is because they have certain concepts (like “not to resuscitate” or “withdraw the treatment”) that are settled and accepted by the society and the laws. However, in countries such as Turkey (predominantly Muslim countries), these concepts do not exist. Therefore, the existing approach is to accept all patients and to care for them until they die, rather than a rational triage. Perhaps, this approach may be one of the reasons why our mortality rates are higher than in European and North American countries.

Scoring systems are significant for estimating mortality risk and identifying the severity of acute organ failure. Some scoring systems that are commonly used in HM patients (as in other ICU patients) are the Acute Physiology and Chronic Health Evaluation (APACHE) II and the Sequential Organ Failure Assessment (SOFA) scores. SOFA has been widely used in ICU patients to assess the organ failures and to identify the sepsis. High SOFA scores or upward trend in SOFA scores over time has been used to predict ICU mortality or to make a decision about the discontinuation of treatment in HM patients. However, using of APACHE II scores to predict prognosis for HM patients have been rather controversial. While some studies have suggested its use for predicting prognosis, others have stated that it is not related with prognosis [4,7,13,29–32]. Hence, none of the relevant scoring systems provides adequately comprehensive data when used alone, they should be used to identify patients at high risk, requiring early, intensive intervention. In this study, we evaluated both APACHE II and SOFA scores for their predictive value. We found that only SOFA scores were predictive for ICU mortality (particularly SOFA score > 8). This conflicts with other similar studies, which report that APACHE II scores between 22 and 25 were independent prognostic factor for ICU mortality [1,3,11–14,25,29].

The number and severity of organ failures seem to be crucial for the prognosis of critically ill HM patients in the ICU. The most common organ failures in ICU patients are pulmonary and renal failure. Due to hemodynamic instability, fluid imbalance, diuretic overuse, sepsis, and nephrotoxic drugs, acute renal failure is more frequent in the ICU. Previous studies have showed that acute renal failure and renal replacement therapy are associated with higher ICU and in-hospital mortality in all patient groups in the ICU, including HM patients [10,22,33]. We had a similar finding here, showing that AKI development during ICU stay was an independent risk factor for ICU mortality. In HM patients, acute pulmonary failure development is associated with much higher mortality risk, particularly when it necessitates invasive mechanical ventilation. Nearly all research in this regard state that IMV is a strong prognostic factor in critically ill HM patients [2,22,34–36]. More than half of all critically ill HM patients need IMV to improve respiratory failure, most resulting in in-hospital or ICU mortality. HM patients who undergo HSCT and need to mechanical ventilation support show mortality rates as high as 96% [37]. This number was similarly higher than overall mortality in our sample, reaching 94.7% versus 51.4%. To avoid intubation and reduce the IMV complications in immunocompromised patients with acute respiratory failure, noninvasive mechanical ventilation (NIV) has been introduced as an alternative. Numerous authors have highlighted that NIV is effective in improving gas exchange abnormalities, reducing endotracheal intubation requirement, and improving outcomes. However, NIV failure (relatively common in critically ill HM patients with acute respiratory failure) is associated with very high mortality. In our study, according to univariable analysis, failure in noninvasive mechanical ventilation and subsequent requirement of invasive mechanical ventilation were more common in nonsurvivors. However, in multivariable analysis, it was not an independent risk factor for ICU mortality. Thus, NIV should be applied early during respiratory failure, with a high alertness for endotracheal intubation in patients without a rapid response [38,39].

Stem cell transplantation can provide long-lasting remission for certain HM patients. After undergoing HSCT, some patients may require ICU admission. These patients (especially after allogeneic HSCT) have a weakened immune system; therefore, they suffer from severe infectious or noninfectious complications, leading to higher ICU mortality rates. With targeted therapy, careful patient selection, and more attentive posttransplantation care, the outcomes of these patients have improved significantly. However, patients especially who undergo allogeneic HSCT and develop severe GVHD or other severe complications still have poor outcomes. With careful patient selection, HSCT patients may have better survival rates and they may benefit from ICU admission [40–42]. According to our findings, undergoing HSCT is not associated with ICU outcome, this may stem from the fact that HSCT patients admitted to our ICU were in the late period of posttransplantation.

The time from the onset of symptoms to ICU admission is an independent predictor of mortality in critically ill patients. Most studies highlight the importance of early ICU admission in critically ill HM patients, although this “early admission” concept is not clearly defined in them. Some authors have considered early admission to be ICU admission directly from the emergency department, whereas others have considered early admission to be ICU admission before multiple organ failure occurs. Here, bed availability is a major determinant in settings with scarce ICU resources. Patients with subtle physiological derangements that might indicate organ failure should be closely monitored to prevent secondary deterioration. It may be dangerous to transfer directly these patients from emergency department to wards [43,44]. In our sample, nonsurvivors were more frequently transferred from hematology wards, stayed longer in the wards, and waited longer for ICU admission. For this patient group, delayed ICU admission and suboptimal treatment in the wards may have led to poor ICU outcomes. These patients are at high risk and require specific management and close monitoring against subtle physiological derangements to foresee organ dysfunctions. Therefore, having an ICU that is dedicated to HM patients (as in our hospital) can help improve their prognosis facilitating early ICU admission, as monitoring may not be optimal in the wards.

Patients with HM are more vulnerable to infection than other patient groups due to the nature of immunosuppression, medications, chemotherapy, and stem cell transplantation, making it a common reason for ICU admission. Previous research reports high ICU mortality rates in HM patients with severe infection (particularly gram-negative sepsis and invasive fungal infection) [45,46]. Besides, HM patients are at high risk of nosocomial infections, which often worsen their outcomes. In our sample, infections and related sepsis/septic shock were the most common causes of ICU admission. ICU-acquired infections (particularly multiresistant microorganisms) were more common among nonsurvivors. We found that developing septic shock during ICU stay due to infections by multiresistant microorganisms was the strongest independent predictor of fatal outcomes, again in agreement with previous research [2,7,27,45,46].

Based on previous studies, some laboratory parameters have been associated with poorer ICU outcomes, such as high levels of lactate, urea, and bilirubin, or low levels of protein, albumin, phosphate, hemoglobin and platelet [2,3,4,10,22]. In our study, we observed higher levels of ALT, LDH, bilirubin, prothrombin time (hepatic dysfunction), urea, and sodium, and lower levels of hemoglobin, platelet, and albumin in nonsurvivors. Although, none of these parameters was significantly prognostic factors for ICU mortality.

This current study has some limitations. Firstly, it is a retrospective, single-center study and has a relatively small study population (368 critically ill HM patients). However, our center is one of the biggest hematology centers in the region and the present study population is not small considering the overall number of HM patients in the previous ICU studies. Secondly, due to some variations in case mix, ICU admission and discharge criteria, intubation rates, and end-of-life decision-making practices, we can hardly compare mortality rates with other research. Thirdly, because of the retrospective nature of the study, some data may have been lost. Finally, since this is a single-center study, our findings cannot be generalized to the entire population. Despite these limitations, we believe that this study is a significant contribution to the growing body of literature on predictors of outcomes for HM patients who are admitted to the ICU.

In conclusion, we evaluated the clinical characteristics, treatments, and ICU outcomes of critically ill HM patients who were admitted to the ICU of a university hospital in Turkey, and we tried to identify risk factors for ICU mortality. We observed a high rate of mortality in our sample. We found multiple potential variables, but only four were independent predictors of ICU mortality. These were high SOFA scores at admission, development of septic shock and acute kidney injury during ICU stay, and invasive mechanical ventilation support during ICU stay. In addition, some variables that were previously associated with poor outcomes like neutropenia, age, underlying diagnosis, and disease status were not found as prognostic factor. There is still a need for further research to better understand poor outcome predictors, admission criteria, treatment, management, and efficient use of resources in critically ill HM patients.

## Figures and Tables

**Table 1:** General characteristics of critically ill HM patients (survivors and nonsurvivors) before and at ICU admission.

Parameters	All HM patients (n = 368)	Survivors (n = 179)	Nonsurvivors (n = 189)	p values
Age* **(year)**	58 (49–67)	58 (52–66)	59 (42–68)	0.636
*< 65 year (n, %)*	253 (68.75)	127 (50.2)	126 (49.8)	0.376
*≥ 65 year (n, %)*	115 (31.25)	52 (45.2)	63 (54.8)	
**Sex** (*male*, *n, %)*	233 (63.3)	121 (67.6)	112 (59.3)	0.097
**APACHE II score at ICU admission** *	23 (18–27)	20 (16–24)	26 (21–31)	**0.0001**
**SOFA score at ICU admission** *	8 (6–11)	7 (5–9)	10 (7–13)	**0.0001**
Duration in hospital before ICU admission* **(day)**	5 (0–24)	3 (2–14)	8 (5–26)	**0.0001**
Waiting time for ICU admission* **(h)**	6 (4–12)	5 (3–10)	8 (4–15)	**0.001**
Length of ICU stay* **(day)**	6 (4–12)	5 (3–10)	8 (4–15)	**0.002**
**Unit before ICU admission (n, %)**				
*Hematology wards*	281 (76.4)	123 (68.7)	158 (83.6)	**0.001**
*Emergency department*	64 (17.4)	41 (22.9)	23 (12.2)	**0.007**
*Others* ¥	23 (6.25)	11 (6.15)	12 (6.34)	0.885
**Hematological malignancies (n, %)**				
*Acute leukemia*	159 (43.2)	74 (41.3)	85 (45)	0.482
*Lymphoma*	95 (25.8)	38 (21.2)	57 (30.2)	0.05
*Multiple myeloma*	108 (29.3)	64 (35.8)	44 (23.3)	**0.009**
*Others* ¶	6 (1.6)	3 (1.7)	3 (1.6)	0.875
**Status of hematological malignancies (n, %)**				
*Newly diagnosed*	127 (34.5)	61 (34.1)	66 (34.9)	0.865
*Complete or partial remission*	64 (17.4)	37 (20.7)	27 (14.3)	0.106
*Recurrence or progression*	147 (39.9)	67 (37.4)	80 (42.3)	0.338
*Terminally ill*	30 (8.15)	4 (2.23)	26 (13.76)	**0.0001**
**Hematopoietic stem cell transplantation (HSCT) (n, %)**				
*Allogeneic*	94 (25.5)	45 (25.1)	49 (25.9)	0.863
Time between HSCT and ICU admission* (day)	210 (48–600)	396.5 (92.25–710)	170 (22–372.4)	**0.016**
*Autologous*	75 (20.4)	43 (24)	32 (16.9)	0.091
Time between HSCT and ICU admission* (day)	425 (144.5–1207.5)	350 (138.75–1000)	720 (150–1460)	0.279
**Reasons for ICU admission (n, %)**				
*Sepsis/septic shock*	277 (75.3)	123 (68.7)	154 (81.5)	**0.005**
*Respiratory*	251 (68.2)	113 (63.1)	138 (73)	**0.042**
*Renal/metabolic*	82 (22.3)	30 (16.8)	52 (27.5)	**0.013**
*Neurological*	78 (21.2)	26 (14.5)	52 (27.5)	**0.002**
**Focus for sepsis at ICU admission (n, %)**				
*Pulmonary*	215 (58.4)	91 (50.8)	124 (65.6)	**0.004**
*Bloodstream/catheter-related bloodstream*	63 (17.1)	27 (15.1)	36 (19)	0.313
*Abdominal*	48 (13)	17 (9.5)	31 (16.4)	**0.049**
*Urinary*	31 (8.4)	10 (5.6)	21 (11.1)	0.057
**Comorbidities (n, %)**				
*Pulmonary*	44 (12)	24 (13.4)	20 (10.6)	0.404
*Cardiological*	90 (24.5)	51 (28.5)	39 (20.6)	0.08
*Neurological*	18 (4.9)	7 (3.9)	11 (5.8)	0.396
*Renal (end stage)*	36 (9.8)	18 (10.1)	18 (9.5)	0.599
*Solid tumors*	17 (4.6)	4 (2.2)	13 (6.9)	**0.034**
**At ICU admission (n, %)**				
*Vasopressor support*	127 (34.5)	54 (30.2)	73 (38.6)	0.103
*Invasive MV support*	85 (23.1)	18 (10.1)	67 (35.4)	**0.001**
*Acute kidney injury*	174 (47.3)	75 (41.9)	99 (52.4)	0.458
*Neutropenia*	146 (39.7)	60 (33.5)	86 (45.5)	**0.016**
**Some laboratory parameters at ICU admission**				
Hemoglobin* *(g/dL)*	8.12 (7.2–9.4)	8.48 (7.3–9.7)	7.8 (7.14–8.91)	**0.009**
*Leukocyte* * *(/mm^3^ )*	4120 (700–9620)	4980 (1040–8800)	3200 (480–10644.5)	0.263
Platelet* *(/mm^3^ )*	42250 (22275–95550)	53000 (26000–124900)	37500 (21000–66500)	**0.002**
Prothrombin time* *(INR)*	1.245 (1.1–1.58)	1.2 (1.08–1.4)	1.325 (1.13–1.67)	**0.001**
CRP* *(mg/L)*	168 (77–265)	159.5 (77.5–246.25)	176 (76.5–286)	0.455
Procalcitonin* *(ng/mL)*	2.5 (0.67–13.11)	2.4 (0.6–17)	2.7 (0.8–11.68)	0.536
BUN* *(mg/dL)*	28 (17.4–48)	23 (16.4–38)	34 (20–54.5)	**0.0001**
Creatinine* *(mg/dL)*	1.08 (0.63–2.08)	1 (0.62–1.9)	1.2 (0.67–2.33)	0.233
Sodium* *(mmol/L)*	137 (133–141)	135 (132–138)	139 (135–144)	**0.0001**
Albumin* *(g/dL)*	2.6 (2.2–2.9)	2.7 (2.35–3)	2.5 (2.1–2.86)	**0.001**
ALT* *(U/L)*	18 (11–39)	17 (11–31)	23 (11–47)	**0.047**
LDH* *(U/L)*	376 (264.3–681)	347.5 (231–496)	445.5 (280.75–834.75)	**0.0001**
Total bilirubin* *(mg/dL)*	0.92 (0.6–2.1)	0.8 (0.5–1.6)	1.17 (0.69–2.78)	**0.0001**
**Vital signs at ICU admission**				
Body temperature* *(°C)*	36.6 (36.38–37)	36.6 (36.4–37)	36.6 (36.3–37)	0.501
Heart rate* *(/min)*	114.5 (100–129.25)	112 (99–126)	120 (102–130.5)	**0.037**
Respiratory rate* *(/min)*	28 (23–32)	28 (22–32)	28 (24–32)	0.369
MAP* *(mmHg)*	73 (64–86)	75 (65–88.5)	72 (62–84)	0.066

* median (25–75 percentiles)

HM: hematological malignancies, APACHE II: Acute Physiology And Chronic Health Evaluation, SOFA: Sequential Organ Failure Assessment, ICU: Intensive care unit, MV: mechanical ventilation, CRP: C-reactive protein, BUN: blood urea nitrogen, ALT: alanine aminotransferase, LDH: lactate dehydrogenase, MAP: mean arterial pressure.

¥ *Other* units before ICU admission were other intensive care units in 8 patients, other in-patient clinics in 11 patients, and other hospitals in 4 patients.

¶ *Other* hematological malignancies were chronic myeloid leukemia in 2 patients, and chronic lymphocytic leukemia in 4 patients.

**Table 2:** General characteristics of critically ill HM patients (all, survivors and nonsurvivors) during ICU stay.

Parameters	All HM patients (n = 368)	Survivors (n = 179)	Nonsurvivors (n = 189)	p values
**MV during ICU stay (n, %)**				
*Invasive MV*	227 (61.7)	48 (26.8)	179 (94.7)	**0.0001**
*Noninvasive MV*	160 (43.5)	79 (44.1)	81 (42.8)	0.805
*Noninvasive MV failure*	103 (28)	27 (15.1)	76 (40.2)	**0.0001**
**Central venous catheterization during ICU stay (n, %)**	273 (74.2)	100 (55.9)	173 (91.5)	**0.0001**
**Acute kidney injury during ICU stay (n,%)**	116 (31.5)	6 (3.4)	110 (58.2)	**0.0001**
**RRT during ICU stay (n, %)**	145 (39.4)	38 (21.2)	107 (56.6)	**0.0001**
*CRRT*	93 (25.3)	8 (4.5)	85 (45)	**0.0001**
**Acquired infections during ICU stay (n, %)**	97 (26.4)	21 (11.7)	76 (40.2)	**0.0001**
*Pneumonia/VAP*	66 (17.9)	13 (7.3)	53 (28)	**0.0001**
*Bloodstream/catheter-related bloodstream*	43 (11.7)	7 (3.9)	36 (19)	**0.0001**
*Urinary*	38 (10.3)	13 (7.3)	25 (13.2)	0.060
**Sepsis/septic shock during ICU stay (n, %)**	123 (33.4)	10 (5.6)	113 (59.8)	**0.0001**
**Cardiac complications during ICU stay (n, %)**	30 (8.2)	9 (5)	21 (11.1)	**0.033**
**GI bleeding during ICU stay (n, %)**	30 (8.2)	4 (2.2)	26 (13.8)	**0.0001**
**Blood/blood products replacement during ICU stay (n, %)**				
*ES*	272 (73.9)	110 (61.5)	162 (85.7)	**0.0001**
*PS*	236 (64.1)	84 (46.9)	152 (80.4)	**0.0001**
*FFP*	95 (25.8)	24 (13.4)	71 (37.6)	**0.0001**
*Albumin*	145 (39.4)	52 (29.1)	93 (49.2)	**0.0001**
Antimicrobial therapy* **during ICU stay (n,%)**				
*Antimicrobials with gram-negative coverage*	353 (95.9)	166 (92.7)	187 (98.9)	**0.008**
*Antimicrobials with gram-positive coverage*	343 (93.2)	160 (89.4)	183 (96.8)	**0.011**
*Antifungal agents*	222 (60.3)	78 (43.6)	144 (76.2)	**0.0001**
*Antiviral agents*	111 (30.2)	45 (25.1)	66 (34.9)	**0.048**
*TMP-SMX for PCP*	97 (26.4)	38 (21.2)	59 (31.2)	**0.035**
*Antimicrobials with anaerobic pathogen coverage*	46 (12.5)	20 (11.2)	26 (13.8)	0.479

HM: hematological malignancies, ICU: intensive care unit, MV: mechanical ventilation, RRT: renal replacement therapy, CRRT: continuous renal replacement therapy, VAP: ventilator associated pneumonia, GI: gastrointestinal, ES: erythrocyte suspension, PS: platelet suspension, FFP: fresh frozen plasma, TMP-SMX: trimethoprim-sulfamethoxazole, PCP: Pneumocystis carinii (jirovecii) pneumonia.

* given according to empirical or definite indications.

**Table 3:** Independent risk factors for ICU mortality in critically ill HM patients according to multivariate analysis.

Parameters	Wald	Significance	Exp (B) or OR	95% CI for OR
APACHE II score at ICU admission	1.311	0.252	1.046	0.968–1.130
SOFA score at ICU admission	8.291	**0.004**	1.281	1.082–1.517
Waiting time for ICU admission (h)	1.534	0.216	1.034	0.981–1.089
Transferring from hematology wards to ICU	0.987	0.321	1.743	0.582–5.214
Admission to ICU due to sepsis	0.178	0.673	0.766	0.223–2.634
Pulmonary focus for sepsis at ICU admission	0.641	0.424	1.503	0.554–4.076
Presence of neutropenia at ICU admission	0.218	0.640	0.804	0.322–2.009
Albumin level at ICU admission	0.234	0.628	0.848	0.436–1.650
LDH level at ICU admission	0.500	0.480	1.000	1.000–1.001
IMV support at ICU admission	2.347	0.125	0.421	0.139–1.273
Vasopressor support at ICU admission	0.577	0.447	0.663	0.230–1.913
IMV support during ICU stay	23.977	**0.0001**	23.118	6.577–81.263
NIV failure	1.452	0.228	0.515	0.175–1.516
Presence of acquired infections during ICU stay	3.148	0.076	0.346	0.107–1.117
Development of septic shock during ICU stay	20.148	**0.0001**	17.123	4.954–59.183
Development of acute kidney injury during ICU stay	30.630	**0.0001**	48.284	12.232–190.594
Antifungal therapy during ICU stay	0.026	0.872	1.081	0.418–2.799
GI bleeding during ICU stay	1.887	0.169	3.592	0.580–22.261

HM: hematological malignancies, APACHE II: Acute Physiology And Chronic Health Evaluation, SOFA: Sequential Organ Failure Assessment, ICU: intensive care unit, LDH: lactate dehydrogenase, IMV: invasive mechanical ventilation, NIV: noninvasive mechanical ventilation, GI: gastrointestinal.
